# Short Peptides from Asian Scorpions: Bioactive Molecules with Promising Therapeutic Potential

**DOI:** 10.3390/toxins17030114

**Published:** 2025-02-28

**Authors:** Kaiyun Xin, Ruize Sun, Wanyang Xiao, Weijie Lu, Chenhui Sun, Jietao Lou, Yanyan Xu, Tianbao Chen, Di Wu, Yitian Gao

**Affiliations:** 1 Zhejiang Provincial Key Laboratory for Water Environment and Marine, Biological Resources Protection, College of Life and Environmental Science, Wenzhou University, Wenzhou 325035, China; 22451335049@stu.wzu.edu.cn (K.X.); 22461338025@stu.wzu.edu.cn (W.X.); 22461338016@stu.wzu.edu.cn (W.L.); 22461338022@stu.wzu.edu.cn (C.S.); 2 School of Pharmaceutical Sciences, Wenzhou Medical University, Wenzhou 325035, China; ljt0818wjy@163.com (J.L.); 18258370103@163.com (Y.X.); 3 Natural Drug Discovery Group, School of Pharmacy, Queen’s University Belfast, Belfast BT7 1NN, UK; rsun03@qub.ac.uk (R.S.); t.chen@qub.ac.uk (T.C.)

**Keywords:** scorpion venom peptides, ion channel blocker, membrane-targeting mechanisms, therapeutic potential, peptide engineering

## Abstract

Scorpion venom peptides, particularly those derived from Asian species, have garnered significant attention, offering therapeutic potential in pain management, cancer, anticoagulation, and infectious diseases. This review provides a comprehensive analysis of scorpion venom peptides, focusing on their roles as voltage-gated sodium (Nav), potassium (Kv), and calcium (Cav) channel modulators. It analyzed Nav1.7 inhibition for analgesia, Kv1.3 blockade for anticancer activity, and membrane disruption for antimicrobial effects. While the low targeting specificity and high toxicity of some scorpion venom peptides pose challenges to their clinical application, recent research has made strides in overcoming these limitations. This review summarizes the latest progress in scorpion venom peptide research, discussing their mechanisms of action, therapeutic potential, and challenges in clinical translation. This work aims to provide new insights and directions for the development of novel therapeutic drugs.

## 1. Introduction

Natural-source medicines have a long history in traditional medicine, with plant- and animal-derived medicines being widely used bioactive substances [1]. For example, the ancient Egyptians and Sumerians used willow bark to relieve pain and inflammation [2]. In addition, *Curcuma longa* is one of the most important traditional herbs in ancient India, primarily used for anti-inflammatory, anticancer, and antioxidant purposes [3]. *Panax ginseng* Meyer, as a traditional medicinal plant, has a long history of use in countries such as China, Korea, and India. Its roots are used to regulate bodily functions, enhance immunity, combat aging, alleviate stress, and treat various symptoms of weakness [4]. However, animal-derived medicines hold a unique position in traditional medicine due to their diverse bioactive components, such as peptides and proteins, and their potential efficacy in treating chronic diseases such as neuropathic pain and rheumatoid diseases [5]. As early as 200-130 B.C., the Greeks utilized leeches for bloodletting treatments to address skin diseases, arthritis, and gynecological disorders [6]. In ancient Rome, the use of animal venom was prevalent in the treatment of various ailments, including smallpox, leprosy, fever, wound healing, and rheumatism [7]. Common sources of animal medicines include bees, scorpions, wasps, spiders, ants, centipedes, frogs, snakes, and toads; notably, scorpions have attracted significant attention in traditional Chinese medicine due to their analgesic and anti-inflammatory properties [8]. The therapeutic use of dried scorpions for treating epilepsy and pain has been documented since the Song Dynasty [9]. With the deepening of research on animal drugs, animal venom has become one of the most important sources of natural products with useful pharmacological activities [10]. Recent advancements in technologies such as proteomics, transcriptomics, and genomics have facilitated the use of various animal venoms in drug development, including those derived from coelenterates (sea anemones), mollusks (cone snails), annelids (leeches), arthropods (scorpions, bees, wasps, spiders, ants, centipedes), amphibians (frogs, toads), and reptiles (snakes, lizards) [11,12,13,14,15,16,17,18]. To date, more than 10 animal venom-derived drugs have received marketing approval, and these drugs address a range of conditions such as chronic pain, type 2 diabetes mellitus, heart failure, and hypertension (Table 1) [19,20]. More than half of them are peptides and peptidomimetics, including captopril, tirofiban, and eptifibatide from snake venom, ziconotide from cone snail venom, lepirudin and bivalirudin from leech venom, and exenatide from lizard venom, among others. Batroxobin, derived from snake venom, is a protein and is commonly classified as a fibrinolytic or thrombolytic drug (Table 1). Notably, ziconotide has been approved by the FDA as a non-opioid analgesic for severe chronic pain, specifically targeting N-type voltage-sensitive calcium channels to block pain signals along the spinal cord. Its analgesic effect is about 1000 times stronger than that of morphine, and it is non-addictive [21]. Therefore, animal venoms are considered a crucial source for the expansion of novel peptide drugs. Peptides derived from scorpion venom exhibit structural diversity and a broad spectrum of pharmacological activities, demonstrating significant efficacy in antibacterial, anticancer, and analgesic effects. Their high targeting capabilities and low toxicity present valuable resources and promising potential for the development of new drugs.

## 2. Previous Research on Peptides Derived from Scorpion Venom

Since the mid-20th century, researchers have been working on the purification of peptides from scorpion venom. Notably, in 1970, Miranda et al. from the Biochemical Laboratory of the Faculty of Medicine in Marseille, France, successfully isolated 11 peptide neurotoxins from the venoms of *Androctonus australis*, *Buthus occitanus tunetanus*, and *Leiurus quinquestriatus*. This foundational work on the structure and function of these neurotoxins has greatly advanced research in the fields of toxicology, pharmacology, and biomedicine [36]. In 1989, Chinese biochemist Zhou Xinhua first reported the isolation and purification of a peptide (named AEP) with antiepileptic activity from the venom of *Buthus martensii Karsch*. Amino acid analysis showed that AEP is a large molecular weight peptide composed of 61 amino acid residues covering 18 types of amino acids [9]. With the rapid development of modern scientific techniques, the isolation and characterization of peptide fractions from scorpion venom have progressed, leading to the discovery of a broader range of scorpion venom peptides. These peptides are classified into two main types: disulfide-bridged peptides (DBPs) and non-disulfide-bridged peptides (NDBPs). The DBP family usually consists of 13–70 amino acids with 3–4 disulfide bonds, and they exhibit diverse biological activities, especially neurotoxicity, by targeting ion channels (Na^+^, Ca^2+^, K^+^, and Cl^−^ channels) on the cell membranes [37]. In contrast, peptides of the NDBP family are generally 13–56 amino acids long, and they are structurally more variable and exhibit a broader range of biological activities, including antimicrobial, anti-inflammatory, immunomodulatory, analgesic, antioxidant, and antiviral activities [38]. Additionally, scorpion venom peptides can be categorized as short-chain and long-chain based on the peptide length. Short-chain peptides typically consist of 30–40 amino acid residues with 3–4 disulfide bonds and primarily act on ion channels (K^+^/Cl^−^/Ca^2+^). Long-chain peptides usually contain 60–70 amino acids and four disulfide bonds, and they predominantly target sodium channels. These long-chain peptides are further classified into α-scorpion toxin and β-scorpion toxin, based on the distinct sites they target on the voltage-gated sodium channels (VGSCs) and their electrophysiological properties [39,40]. The α-scorpion toxin binds to site 3 of VGSCs, slowing or inhibiting channel deactivation, thus prolonging the action potential of these ion channels. The β-scorpion toxin binds to receptor site 4 of the VGSCs, typically shifting activation voltage dependently to a higher hyperpolarizing potential and reducing peak current amplitude [41].

## 3. Pharmacological Activity of Scorpion Venom Peptides

Scorpion venom peptides demonstrate a wide range of pharmacological activities, including anticancer, antibacterial, analgesic, and anticoagulant activities. The majority of these peptides, specifically the neurotoxic peptides, belong to the DBP family. They function primarily by inhibiting cellular electrophysiological activity through the blockage of ion channels. Thus, scorpion venom peptides are regarded as promising candidates in the development of lead drugs for the treatment of pain and other ion channel-related disorders. In addition to DBPs, certain NDBPs are structurally simpler, yet still exhibit diverse biological functions. Such peptides also enhance the potential applications of scorpion venom peptides in drug discovery and development, paving the way for innovative therapeutic options.

### 3.1. Analgesic Activity of Scorpion Venom Peptides

**Figure 1:**
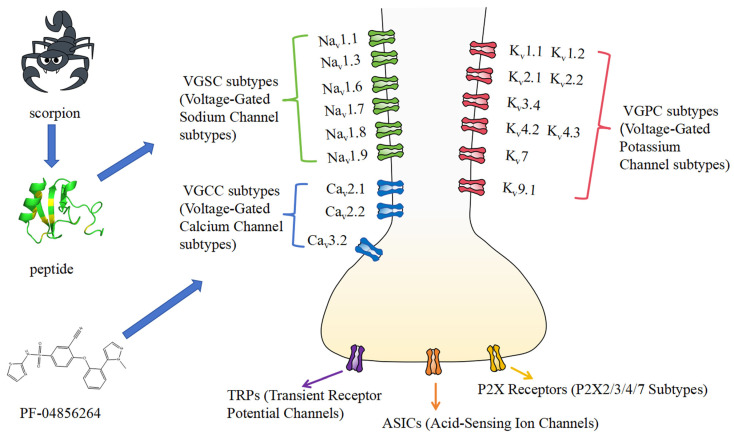
Potential targets of scorpion venom peptides in pain management. Scorpion venom peptides exert their analgesic effects primarily by modulating pain-related ion channels, including VGSCs (green), VGPCs (red), VGCCs (blue), TRPs (purple), purinergic P2X ion channels (yellow), and ASICs (orange).

Scorpion venom peptides exert analgesic effects primarily by modulating pain-related ion channels, including VGSCs, voltage-gated potassium channels (VGPCs), voltage-gated calcium channels (VGCCs), transient receptor potential channels (TRPs), purinergic P2X ion channels, and acid-sensing ion channels (ASICs) (Figure 1). Notably, scorpion venom peptides have been reported to exert significant analgesic effects mainly through their action on VGSC [42]. As a result of their abundant availability and notable analgesic effects, scorpion venom peptides are a potential source of novel analgesic medications. Comprehensive studies on scorpion venom peptides and their mechanisms of action are anticipated to lead to breakthroughs and new therapeutic options in pain management.

#### 3.1.1. VGSCs, VGPCs, and Pain

**Figure 2:**
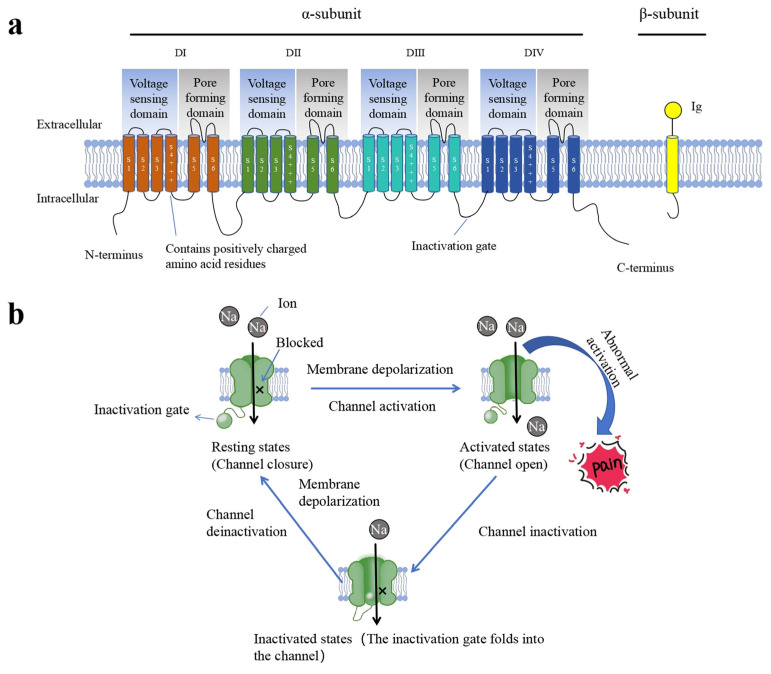
Structural characteristics of VGSCs. (**a**) In mammals, sodium channels are composed of an α-subunit and one or more β-subunits. The α-subunit consists of four homologous domains: DI (orange), DII (green), DIII (turquoise blue), and DIV (blue). Each domain is made up of six transmembrane segments (S1–S6), where S1–S4 form the voltage-sensing domain and S5–S6 constitute the pore-forming domain. The positively charged amino acid residues on the S4 segment detect changes in membrane potential and regulate the opening of the sodium channel. The intracellular loop between DIII and DIV functions as an inactivation gate, closing the sodium channel during rapid inactivation. The β subunit (yellow) consists of an N-terminal extracellular immunoglobulin (Ig)-like fold, a transmembrane segment, and a short intracellular C-terminus. It is an auxiliary component of the VGSC and does not participate in the formation of the pore. (**b**) Sodium ion channels exist in three states: the resting state, the activated state, and the inactivated state. When in the activated state, the channel opens, allowing the influx of sodium ions. Abnormal activation of sodium ion channels can lead to the generation of pain responses in the body.

The VGSCs in eukaryotes consist of an α-subunit and multiple auxiliary β-subunits (Figure 2). The α-subunit is a single-stranded transmembrane glycoprotein encoded by the *SCN9A* gene, comprising four homologous structural domains (DI, DII, DIII, and DIV). Each domain contains six transmembrane helical segments (S1–S6), where S1–S4 form the voltage-sensing domain (VSD), and S5, S6, and the loops (pore-loops, P-loops) between them form the pore domain (PD). When the cell membrane is in a depolarized state, the positively charged amino acid residues (lysine and arginine residues) on the S4 segment can detect changes in the membrane potential. This leads to the movement of the S1, S2, and S3 segments towards the extracellular surface, resulting in a conformational change in the α-subunit that regulates the opening of the sodium ion channel [43]. In contrast, during depolarization, an inactivation gate within an intracellular loop of the homologous DIII and DIV domains acts like a hinge lid, rapidly inactivating the sodium channel by folding into the pore [43]. Each β-subunit of VGSC comprises an N-terminal extracellular immunoglobulin (Ig)-like fold, a single transmembrane segment, and a short intracellular C-terminus. These β-subunits (including the β1, β1B, β2, β3, and β4 isoforms) are auxiliary components of the VGSC and do not contribute to pore formation [44]. Among them, β1 or β3 binds to the α-subunit non-covalently, while β2 or β4 binds to the α-subunit through disulfide bonds. The different β-subunits of VGSC play important roles in regulating the expression of the α-subunit, channel activation and inactivation, voltage-dependent gating, and the expression of channels on the cell surface [45].

Mammals have nine different α-subunits, and these correspond to nine VGSC subtypes (Nav1.1 to Nav1.9). The expression of several subtypes in the peripheral nervous system (Nav1.1, Nav1.3, Nav1.6, Nav1.7, Nav1.8, and Nav1.9) is critical for pain signaling, especially Nav1.7, Nav1.8, and Nav1.9 [46]. Genetic and physiological studies indicate that mutations in *SCN9A* (Nav1.7), *SCN10A* (Nav1.8), and *SCN11A* (Nav1.9) can lead to abnormal pain sensitivity in humans [47]. Loss-of-function mutations in *SCN9A* will lead to congenital insensitivity to pain syndrome, while gain-of-function mutations often cause neuropathic pain conditions in humans, such as erythermalgia and paroxysmal extreme pain disorder. In addition, elevated Nav1.7 expression is strongly related to the development of diabetes and inflammatory pain [48]. Gain-of-function mutations in both *SCN10A* and *SCN11A* have been identified in patients suffering from painful peripheral neuropathy.

Voltage-gated potassium channels and sodium ion channels share many structural similarities but also exhibit significant differences. Both channels are composed of a functional α subunit and an auxiliary β subunit, with six transmembrane domains labeled S1 to S6. The S1 to S4 regions form the voltage-sensing domain, and the pore is formed by the angle loop between S5 and S6 [49]. In contrast, voltage-gated potassium channels are composed of four independent polypeptide subunits, forming a heterotetramer, which may exhibit different time-dependent and voltage-dependent properties [50]. Based on the amino acid sequence of the protein’s hydrophobic core, Kv channels are classified into 12 subfamilies (Kv1 to Kv12), with a total of 40 distinct Kv channel subunits. These channels play a crucial role in action potential repolarization and the suppression of membrane depolarization, which is fundamental to regulating neuronal excitability. Dysfunction or downregulation of potassium channels is associated with neuronal hyperexcitability disorders such as epilepsy and neuropathic pain. For instance, Chien et al. found that in a neuropathic pain model induced by spinal nerve ligation in rats, the protein levels of Kv3.4 and Kv4.3 in the DRG neurons were greatly reduced [51]. The opening of K^+^ channels leads to membrane hyperpolarization, thereby reducing cellular excitability. As a result, certain Kv channels are considered potential candidate targets for pain treatment. In particular, drugs targeting the Kv1 and Kv7 families may become promising candidates for the treatment of neuropathic pain [52]. For example, in peripheral nerve injury models induced by lumbar 5 spinal nerve ligation (SNL) and sciatic nerve axotomy, the expression of Kv1.2 in DRG neurons is downregulated, and this downregulation can be restored by overexpressing DRG Kv1.2 RNA, thereby preventing the development and maintenance of SNL-induced neuropathic pain [53].

#### 3.1.2. Blockers Targeting VGSCs and Activators Targeting VGPCs for the Treatment of Pain

Sodium channel blockers are a class of medications that affect the excitability of nerve and muscle cells by binding to sodium channels and preventing them from opening, thereby reducing or blocking the inward flow of sodium ions. These drugs are often used to treat epilepsy, cardiac arrhythmias, and chronic pain. Common sodium channel blockers currently in clinical practice include carbamazepine, lidocaine, procaine, oxcarbazepine, and quinidine (Table 2) [54,55,56,57,58,59]. However, the effectiveness of these traditional drugs in pain management is often limited, and the adverse effects may restrict their clinical use. For example, although lidocaine can alleviate symptoms in patients with chronic neuropathic pain through multiple delivery modes, it poses risks of liver damage, along with potential adverse reactions and toxic side effects, and has strict dose limits [60,61]. Carbamazepine is currently the preferred oral drug used to control paroxysmal pain in patients with trigeminal neuralgia. However, carbamazepine works slowly and causes multiple side effects, such as dizziness and elevated aminotransferases [62]. Therefore, there is an urgent need to develop new, efficient, and safer drugs for the treatment of chronic pain.

Currently, compounds such as AZD3161, CNV1014802, PF-05089771, XEN402, and PF-04856264 are being developed into Nav1.7 channel blockers, and their development has reached the clinical level (Table 2) [63,64,65]. Among them, the administering of PF-04856264, both topically and systemically, has been shown to exhibit high selectivity in its inhibition of Nav1.7, effectively reversing the scorpion venom OD1-induced spontaneous pain behaviors [64]. Notably, it does not cause significant adverse motor reactions and toxicities, even at a high dose (30 mg/kg). Besides the examples given, others, such as VX-548, PF-04531083, and 2j (VX-150-derived compounds) have also been found to be analgesic compounds that specifically target Nav1.8 channels [66,67]. Compound 2j can effectively inhibit Nav1.8 channels and shows significant analgesic effects in the Complete Freund’s Adjuvant (CFA)-induced chronic inflammatory pain model and the chronic constriction injury model (CCI) in mice [67]. VX-548 produced a significantly higher level of analgesic effect than a placebo in abdominoplasty and bunionectomy trials involving 303 and 274 volunteers, respectively, and demonstrates favorable safety, tolerability, and non-addictive properties [66]. In addition, there are several investigational drugs targeting multiple VGSC subtypes (e.g., Lacosamide and Vixotrigine) that have demonstrated good efficacy during the clinical testing phase [68,69,70]. Although these novel VGSCs-targeting drugs have exhibited great analgesic potential in clinical trials, further evaluation of their safety and efficacy is still required.

In contrast, the activation of Kv channels helps inhibit neuronal hyperexcitability and reduce pain perception. Currently, openers of Kv1 and Kv7 (KCNQ) channels have been studied for the treatment of neuropathic pain. By modulating the function of these ion channels, neuronal excitability can be effectively regulated, thereby alleviating pain [52]. For example, Jorge et al. recently demonstrated that ICA-069673 and ML213 are specific Kv7 channel activators that exhibit analgesic effects in an in vitro spinal cord preparation, though they have not yet entered clinical trials [71]. Compared to flupirtine, derivative compound 16 demonstrates higher efficacy in activating K_V_7.2/7.3 channels and successfully avoids the formation of diimine metabolites, thus reducing potential liver damage [72]. Additionally, compound 16 exhibited significant dose-dependent analgesic effects in mouse models of chronic constriction injury and streptozotocin-induced diabetic peripheral neuropathy. These results suggest that compound 16 has potential as a candidate drug for the treatment of neuropathic pain. However, many existing small molecule compounds face several challenges in preclinical and clinical trials, including low bioavailability, poor selectivity, and issues related to efficacy and safety during long-term use, which still need to be addressed.

#### 3.1.3. Scorpion Venom Peptides Targeting VGSCs and VGPCs for the Treatment of Pain

**Figure 3:**
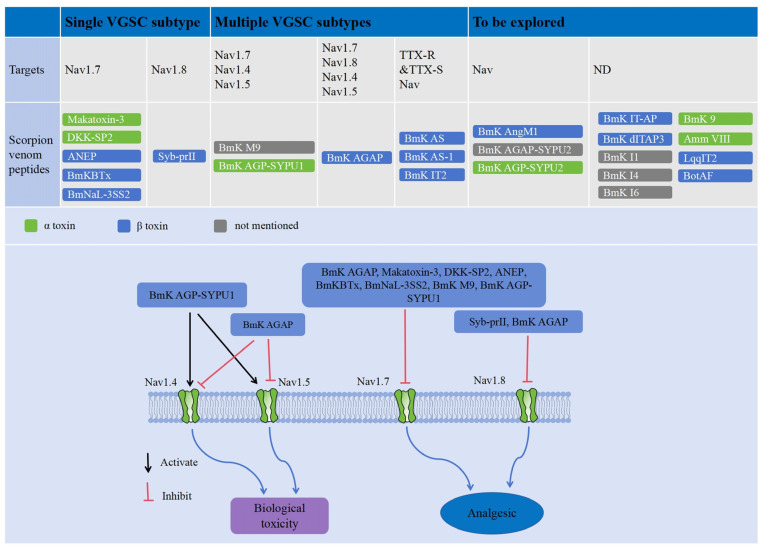
Scorpion venom peptides with analgesic activity and their modes of action. Analgesic scorpion venom peptides targeting sodium channels are divided into three categories: (1) those targeting a single sodium channel, (2) those targeting multiple sodium channels, and (3) those that have not been fully explored. These peptides are further classified into α-toxin (green) and β-toxin (blue), based on distinct action sites on VGSCs and their electrophysiological properties. The scorpion venom peptides that have not yet been clearly classified are represented in gray.

Currently, several scorpion venom peptides that target VGSC subtypes and exhibit unique analgesic activity have been identified. For example, the scorpion venom peptides Makatoxin-3, anti-neuroexcitation peptide (ANEP), DKK-SP2, BmKBTx, and BmNaL-3SS2, all of which are derived from *Buthus martensii Karsch*, can exert significant analgesic effects in acute inflammatory pain mainly by inhibiting the VGSC subtype Nav1.7 (Figure 3 and Table A1) [73,74,75,76,77,78]. Notably, in an acetic acid-induced mouse model of acute inflammatory pain, BmNaL-3SS2 was found to exhibit greater analgesic effects than morphine, while DKK-SP2 and BmKBTx were slightly less analgesic than morphine. Makatoxin-3 is more potent than Nav1.7-selective inhibitors and nonsteroidal anti-inflammatory drugs (NSAIDs) in formalin animal models. Whole-cell patch clamp tests have shown that DKK-SP2, BmKBTx, and BmNaL-3SS2 inhibit Nav1.7 currents in hNav1.7-CHO cells in a dose-dependent manner. Additionally, when tested in a rat infraorbital nerve chronic constriction injury (IoN-CCI) model, DKK-SP2 was found to significantly reduce the expression of Nav1.7 in trigeminal ganglion (TG) neurons, resulting in the relief of chronic neuropathic pain. Tests carried out with Makatoxin-3 also showed that it can exert significant analgesic effects in a mouse model of chronic inflammatory pain induced by CFA.

In addition, the neurotoxin scorpion venom peptide Syb-prII and its mutants Syb-prII-1 and Syb-prII-2, which primarily target the Nav1.8 ion channel, can significantly alleviate formalin-induced acute inflammatory pain in mice (Figure 3 and Table A1) [79,80]. In particular, the analgesic effect produced by 2.0 mg/kg Syb-prII-1 is comparable to that of 200 mg/kg aspirin [79]. Additionally, Syb-prII-1 can significantly inhibit both Nav1.8 expression and current density when tested against a rat model of chronic neuropathic pain induced by IoN-CCI, and this further illustrates its therapeutic potential [80].

Besides the specific target of VGSCs with analgesic activity, most scorpion venom peptides exhibit activity toward multiple VGSC subtypes, contributing to their analgesic effects. For example, BmK AS, BmK AS-1, and BmK IT2 can reduce the currents of tetrodotoxin-sensitive (TTX-S) and tetrodotoxin-resistant (TTX-R) sodium channels in rat DRG neurons in a dose-dependent manner, thus demonstrating a significant analgesic effect (Figure 3 and Table A1) [81,82,83,84]. In contrast, BmK M9, BmK AGAP, and BmK AGP-SYPU1 can not only modulate Nav1.7 to exert their analgesic activity but also act on Nav1.4 and Nav1.5 channel proteins, which can in turn lead to toxicity in the skeletal and cardiac muscles (Figure 3 and Table A1) [85,86,87,88,89,90]. The analgesic effect of BmK AGAP is also related to the inhibition of Nav1.8, and the core structural domain consisting of G17, R18, W38, and N44 is the key domain linked to the biological activity and toxicity of BmK AGAP [86]. Through targeted mutagenesis, where Trp38 in BmK AGAP was converted to Gly, inhibition of the peak currents of hNav1.4 and hNav1.5 channels by the mutant BmK AGAP is largely attenuated, and this in turn can reduce the toxicity to skeletal and cardiac muscle while preserving analgesic activity comparable to that of wild-type BmK AGAP [87]. This suggests that BmK AGAP-W38G may serve as a safer alternative for clinical applications. In addition, the replacement of Tyr5 and Tyr42 in BmK AGP-SYPU1 with a hydrophobic or hydrophilic amino acid has resulted in a significant increase in the analgesic effect of the mutant peptides. The Y5F mutant, in particular, displays low activation of Nav1.4 and Nav1.5, positioning it as a promising candidate for clinical application due to its enhanced analgesic properties with minimal skeletal and cardiac toxicity [89,90,91,92].

Scorpion venom-derived peptides with long chains have been demonstrated to exhibit analgesic activity in various animal pain models, though their exact targets have not been identified. For example, the peptides BmK IT-AP (72 amino acids), BmK dITAP3 (61 amino acids), BmK AngM1 (64 amino acids), BmK I1 (72 amino acids), BmK I4 (72 amino acids), BmK I6 (61 amino acids), BmK 9 (65 amino acids), BmK AGAP-SYPU2 (65 amino acids), and BmK AGP-SYPU2 (66 amino acids) purified from the venom of *Buthus martensii Karsch* display significant analgesic efficacy in the acetic acid-induced writhing test in mice (Figure 3 and Table A1) [93,94,95,96,97,98,99,100,101]. Among them, BmK AngM1, BmK AGP-SYPU2, and BmK AGAP-SYPU2 can also inhibit sodium currents as demonstrated by electrophysiological experiments, indicating a potential mechanism of action. BmK AGP-SYPU2 and BmK AGAP-SYPU2 are similar in sequence to the antitumor-analgesic peptide BmK AGAP, suggesting that their analgesic effects may be primarily attributed to the blockade of VGSC. Similarly, other long-chain scorpion venom peptides, Amm VIII, LqqIT2, and BotAF from *Androctonus mauretanicus mauretanicus*, *Leiurus quinquestriatus quinquestriatus,* and *Buthus occitanus tunetanus*, respectively, have also been shown to have significant analgesic activity in animal pain models (Figure 3 and Table A1) [102,103,104]. While it is well-speculated that long-chain scorpion venom peptides may exert their analgesic effects through interactions with VGSCs, further research is necessary to elucidate the specific mechanisms underlying their pain-relieving properties.

In addition, the peptides CeII8 and IMe-AGAP, derived from the venom of *Centruroides elegans* and *Mesobuthus eupeus*, respectively, can act on VGSCs, while the specific actions still need to be validated in animal models (Figure 3 and Table A1) [105,106]. CeII8 functions as a blocker of Nav1.7 by inhibiting peak sodium currents, while IMe-AGAP may interact with the structural domains of Nav1.8 and Nav1.9, as shown by computer simulation studies, making it a potential analgesic candidate. However, the analgesic efficacy of CeII8 and IMe-AGAP, as well as the specific mechanisms underlying their biological activities, require further experimental investigation.

Currently, research on scorpion venom peptides that target potassium ion channels and possess analgesic activity is still in the developmental stage. Although most scorpion venom peptides’ analgesic effects are primarily focused on sodium ion channels, some peptides have also demonstrated activity on potassium ion channels. For example, the peptide Hetlaxin isolated from the venom of the scorpion *Heterometrus laoticus* was found to exert antinociceptive effects by binding to the extracellular vestibule of the K^+^-conducting pore of Kv1.1 and Kv1.3 potassium channels [107]. Additionally, another short-chain peptide, BmP02, isolated from the venom of *Buthus martensi Karsch*, can dose-dependently delay the inactivation of the Kv4.2 channel. As an activator of Kv4.2, BmP02 may exhibit analgesic activity, but its efficacy and underlying mechanisms require further investigation [108]. In summary, while the research on scorpion venom peptides targeting potassium ion channels for analgesic activity is still in its early stages, these findings suggest that such peptides could offer promising new avenues for pain management, warranting further investigation into their mechanisms and therapeutic potential.

### 3.2. Antibacterial Activity of Scorpion Venom Peptides

In recent years, many natural peptide compounds derived from scorpion venom have been reported to exhibit strong antibacterial activity. Most of these peptides belong to the NDBP family and typically display cationic amphipathicity. They primarily disrupt the bacterial cell membrane structure through electrostatic attraction, leading to leakage of cellular contents and ultimately causing bacterial death. Although these antimicrobial peptides offer advantages over antibiotics in terms of antibiotic resistance, their lack of high selectivity for bacterial cell membranes often results in strong hemolytic activity and ineffective in vivo performance. This poses significant challenges for the widespread clinical application of natural scorpion venom peptides as antimicrobial agents. Notable examples of such peptides include TtAP-1, Im-5, UyCT3 and UyCT5, Pantinin-3, Meucin-18, VmCT1, BmKn2, and Hp1404, which are derived from the venoms of *Tityus trinitatis*, *Isometrus maculatus*, *Urodacus yaschenkoi*, *Pandinus imperator*, *Mesobuthus eupeus*, *Vaejovis mexicanus smithi*, *Buthus martensii Karsch*, and *Heterometrus petersii*, respectively, and all are active against a wide range of drug-resistant bacteria (Table 3) [109,110,111,112,113,114,115,116]. The lack of selectivity against bacterial cells means that these peptides can cause significant hemolytic activity in mammalian cells. This drawback poses a challenge for the clinical application of natural scorpion venom peptides as antimicrobial agents. For example, BmKn2 has been shown to exhibit potent antibacterial activity against both Gram-positive and Gram-negative bacteria (including drug-resistant strains), with MIC values ranging from 0.6 to 21.3 µg/mL. However, at a concentration of 17.13 µg/mL, it causes 50% hemolysis of human red blood cells [117,118]. To address this problem, Cao et al. increased the number of basic residues on the hydrophilic face of BmKn2-7 by using lysine and arginine, thereby increasing the net charge, and obtained the mutant Kn2-7. Compared to BmKn2, Kn2-7 enhanced its antibacterial activity and reduced hemolytic activity by approximately fivefold (HC_50_ = 90.27 µg/mL). Mechanistic studies showed that Kn2-7 was tightly bound to lipoteichoic acid (LTA), leading to membrane disruption and bacterial lysis, which rapidly killed *S. aureus*. This suggests that increasing the number of basic residues on the hydrophilic face can fine-tune the balance between antimicrobial efficacy and cytotoxicity. This modification enhances the electrostatic interaction between the peptide and the bacterial cell membrane, leading to bacterial lysis while minimizing interaction with the red blood cell membrane, thereby reducing hemolytic effects. Luo et al. further replaced the arginine residues on the hydrophilic face of Kn2-7 with lysine residues, resulting in the derivative peptide Kn2-7K [119]. It was found that the hemolytic activity was minimized, and the peptide exhibited the ability to combat drug-resistant ESKAPE pathogens. This indicates that the electrostatic interaction between the peptide and the bacterial cell membrane surface may be a key determinant of its antibacterial activity. Moreover, by increasing the number of basic residues on the hydrophilic face, the hydrophobicity was reduced, which weakened the hydrophobic interaction between the peptide and the red blood cell membrane, potentially achieving a reduction in hemolytic activity. Similarly, Kim et al. reported that peptide Hp1404-T1e, a mutant peptide derived from Hp1404, has a significantly reduced hemolytic effect on mammalian cells as a result of its enhanced hydrophobic moment and net charge compared with the original peptide Hp1404 [120]. These investigators also showed that Hp1404-T1e exhibits significantly lower cellular toxicity than Hp1404 as demonstrated in MTT assays. Hp1404-T1e exhibits strong antibacterial and antibiofilm activity against multidrug-resistant *Pseudomonas aeruginosa* (MRPA) strains. It has greater stability under a high salt concentration and in the presence of trypsin. Optimizing the hydrophobicity and net charge of peptides is an effective strategy to enhance their therapeutic potential while minimizing toxicity. The drug resistance assay data show that *S. aureus* developed resistance after two treatments with kanamycin (MIC: 6.25 µg/mL), with a 32-fold increase in MIC after nine treatments. In contrast, the MIC of Hp1404 remained unchanged even after 15 treatments, indicating that it is difficult for *S. aureus* to develop resistance to Hp1404 [116]. Mechanistic studies revealed a difference between Hp1404 and Hp1404-T1e in their effects on bacterial cell membranes. While Hp1404 kills bacteria by disrupting the membrane, Hp1404-T1e not only enhances its binding ability to lipopolysaccharides (LPS) but may also enter bacterial cells to act on DNA [120]. However, studies have shown that Bacteria can develop resistance to certain antimicrobial peptides through mechanisms such as modification of the cell membrane, activation of efflux pump systems, biofilm formation, and protease degradation [121,122,123]. It has been reported that the human antimicrobial peptide LL37 and the insect antimicrobial peptide Cecropin B both develop varying degrees of resistance against *S. aureus*, *Salmonella typhimurium*, *Clostridium difficile*, and fish bacterial pathogens [124,125,126,127]. Although few antimicrobial peptides can induce bacterial resistance, compared with traditional antibiotics, scorpion venom peptides still exhibit a lower tendency to induce resistance and have promising application prospects [128].

In addition, Heteroscorpine-1 has gained attention for its antimicrobial properties, particularly due to its N-terminal cecropin-like sequence [130]. Previous studies have shown that the antimicrobial activity of cecropin is closely related to the alanine–glycine–proline hinge region, and removing this hinge region leads to a decrease in antimicrobial activity, although anti-inflammatory effects are improved [132]. Based on these insights, Rima Erviana and colleagues used the cecropin-like region of Heteroscorpine-1 (CeHS-1) as a peptide template, with cecropin B serving as the ideal structure for antimicrobial peptides [131]. By adding a glycine–proline hinge region and removing negatively charged amino acids (such as glutamic acid and aspartic acid), mutant peptides CeHS-1 GP and CeHS-1 GPK were generated. Notably, CeHS-1 GPK involved the substitution of asparagine with lysine at position 19, further increasing the peptide’s positive charge. Through sequence modification, the physicochemical properties were optimized, and the antimicrobial activity of CeHS-1 GPK was significantly enhanced. In a concentration-dependent manner, it was able to disrupt both the inner and outer bacterial membranes without significantly increasing toxicity. Furthermore, combining antimicrobial peptides with conventional antibiotics may be an effective approach to overcoming resistance and improving therapeutic efficacy. The study found that CeHS-1 GPK may promote bacterial uptake of kanamycin by increasing membrane permeability, thereby enhancing the antimicrobial effect [131]. Collectively, These findings highlight the potential of scorpion venom peptides as a source of new antimicrobial agents with tailored properties to reduce toxicity and enhance efficacy. By carefully optimizing peptide structure and charge distribution, as well as exploring novel drug delivery strategies such as targeted delivery systems or nanoparticle formulations, the therapeutic application of scorpion venom peptides could be further advanced for treating bacterial infections. Moreover, the continued study of peptide resistance mechanisms and the design of peptides with lower tendencies to induce bacterial resistance will be essential to ensuring their sustainable clinical application. Combining antimicrobial peptides with antibiotics not only enhances antimicrobial activity but also serves as an effective strategy to overcome existing resistance mechanisms and improve therapeutic outcomes.

Additionally, some scorpion venom peptides, rich in cysteine, exhibit dual functions of antimicrobial activity and ion channel targeting. Their antimicrobial mechanisms differ from the traditional membrane disruption mechanism. For example, the mechanisms of action of Bactridine 1 and analgesic peptides are similar, both involving the regulation of ion selectivity across membranes (Table 3) [129]. Notably, Bactridine 1 can alter the membrane permeability of *Yersinia enterocolitica* at just 1 µM, allowing the selective passage of sodium ions through the cell membrane [129]. Moreover, Bactridine 1 has no effect on host cells, which may be due to the significant differences between bacterial sodium channels and those on eukaryotic cells. Thus, antimicrobial drugs designed to target bacterial sodium channels could potentially represent a safe and effective novel therapy for treating bacterial infections in mammals.

### 3.3. Anticancer Activity of Scorpion Venom Peptides

Extensive research has demonstrated that scorpion venom peptides exert significant analgesic effects primarily through targeting ion channels, and furthermore, ion channels are also closely related to the growth and development of tumor cells. Scorpion venom peptides can inhibit cancer cell proliferation by blocking sodium, potassium, and chloride ion channels. In addition, the membrane disruption mechanism underlying the major mode of action by which antibacterial peptides work has also garnered widespread attention in cancer research.

Although scorpion venom peptides act on ion channels in their analgesic and anticancer effects, the specific mechanisms and signaling pathways differ significantly among various cell types. In terms of their analgesic effects, scorpion venom peptides primarily alleviate pain by blocking ion channels and inhibiting the electrophysiological activity of neurons. In contrast, the mechanisms underlying their anticancer effects are more complex and involve multiple physiological processes, including proliferation, migration, and apoptosis of the cancer cells. For example, the scorpion venom peptide BmK AGAP alleviates pain by suppressing sodium channel currents in DRG neurons, reducing membrane excitability, and blocking pain signal transmission [133]. BmK AGAP inhibits Nav1.5 and further downregulates PTX3 via the NF-kB and Wnt/β-catenin signaling pathways, thereby suppressing stemness, epithelial–mesenchymal transition, migration, and invasion of breast cancer cells (Table 4). Margatoxin, a scorpion venom peptide derived from *Centruroides margaritatus*, inhibits Kv1.3, subsequently impeding the normal transition from the G1 phase to the S phase in the A549 cell cycle, ultimately suppressing the proliferation of lung cancer cells (Table 4) [134,135,136]. Additionally, BmKCT, a scorpion venom peptide from *Buthus martensii Karsch*, specifically targets chloride channels in glioma cells and downregulates MMP-2 expression, thereby inhibiting proliferation, invasion, and migration of glioma cells C6 and U251 (glioma) cells (Table 4) [137,138].

Scorpion venom peptides can exert anticancer effects through mechanisms similar to those involved in antimicrobial peptide-induced membrane disruption. This is primarily because both cancer cell membranes and bacterial cell membranes contain a substantial amount of negatively charged phospholipids. Scorpion venom peptides can target cancer cell membranes through electrostatic interactions, leading to pore formation or membrane rupture, ultimately resulting in cancer cell death. For instance, peptides Smp24 and Smp43 isolated from *Scorpio maurus palmatus* venom induce apoptosis, autophagy, necrosis, and cell-cycle arrest in HepG2 (hepatoma) cells by disrupting cell membrane integrity and mitochondrial function, thus demonstrating significant antitumor activity (Table 4) [140,144]. Additionally, the scorpion venom peptide Pantinin from *Pandinus imperator* preferentially interacts with the negatively charged phospholipids on cancer cell membranes, compromising membrane integrity and showing anticancer activity against MDA-MB-231 (breast cancer) and DU-145 (prostate cancer) cells (Table 4) [145]. Moreover, similar to antimicrobial peptides (AMPs), the activity of anticancer peptides is influenced by factors such as net charge, hydrophobicity, and amphiphilicity. For example, the cationic-enhanced analogs of scorpion venom peptides TsAP-1 and TsAP-2 from *Tityus serrulatus,* and AaeAP1 and AaeAP2 from *Androctonus aeneas* exhibit dose-dependent antiproliferative effects on human cancer cells such as MCF-7 and PC3 cells (Table 4) [146,147]. The increase in net positive charge likely enhances the interaction with target cell membranes, providing new avenues for cancer treatment.

### 3.4. Anticoagulant Activity of Scorpion Venom Peptides

Recent studies have revealed that scorpion venom peptides can inhibit coagulation. Specifically, peptides TanP, P8(HA18-3-B-8), LeuTrp, and IIeTrp, isolated from *Tityus stigmurus*, *Buthus martensii Karsch*, and *Heterometrus laoticus*, respectively, have been shown to possess anticoagulant properties (Table 5) [148,149,150]. Notably, TanP exhibits significant in vitro anticoagulant activity at concentrations of 12.5 and 25 µM [148]. In contrast, LeuTrp and IieTrp do not demonstrate any in vitro anticoagulant activity in human plasma, even at concentrations of up to 100 µM. However, they can significantly prolong tail bleeding time and in vitro clotting time in mice, suggesting their anticoagulant effects might be mediated by inhibiting platelet function [150]. The mechanisms underlying the anticoagulant activity of these dipeptides require further investigation.

## 4. Conclusions

The research history of scorpion venom peptides has been rich and fruitful. These peptides not only have potent biological functions such as antibacterial, anticancer, analgesic, and anticoagulant activities but also demonstrate great potential in the development of new drugs. In particular, scorpion venom peptides target ion channels, demonstrating high selectivity and specificity in treating diseases such as pain and cancer, thereby enhancing efficacy and reducing toxicity. In terms of clinical applications, chlorotoxins have entered clinical trials, and their potential in cancer therapy has been demonstrated in phase II clinical trials, pointing to the feasibility of scorpion venom peptides in clinical applications and laying the foundation for more scorpion venom peptides to enter clinical trials in the future. Scorpion venom is rich in peptides, and more and more scorpion venom peptides will be isolated, purified, and characterized with the further development of genomics, transcriptomics, and proteomics, leading to broader prospects for the research and application of scorpion peptides. All this will serve as a basis for the design and development of new effective drugs and new strategies for the treatment of diseases. However, existing research limitations, such as cytotoxicity, stability, targeting specificity, and challenges in clinical translation, still need to be addressed. Future research should focus on optimizing the efficacy and safety of scorpion venom peptides through molecular engineering and drug delivery systems, particularly in enhancing targeting specificity and reducing off-target cytotoxicity. Additionally, exploring the combination of scorpion venom peptides with other therapeutic strategies may provide new breakthroughs for clinical treatment.

## Figures and Tables

**Table 1:** Approved drugs for human use.

Molecule	Species Origin of Venom Toxin	Production	Structure or Sequence	Use	Developing Company	References
Captopril	*Bothrops jararaca*	Synthetic	[2S]-1-[3-mercapto-2-methyl-propionyl]-L-proline	Hypertension	Bristol-Myers Squibb	[22,23]
Enalapril	*Bothrops jararaca*	Synthetic	[S]-1-[N-(1-[ethoxycarbonyl]-3-phenylpropyl)-L-alanyl]-L-proline	Hypertension	Merck	[22,23]
Tirofiban	*Echis carinatus*	Synthetic	N-(butylsulfonyl)-O-[4-(4-piperidinyl) butyl]-L-tyrosine	Acute coronary syndrome	Merck	[24,25]
Eptifibatide	*Sistrurus miliarius*	Synthetic	CRGDWPC	Acute coronary syndrome		[26,27]
Batroxobin	*Bothrops moojeni*	Purified from venom	231 amino acids	Anticoagulant		[28,29]
Cobratide	*Naja atra*	Purified from venom	LECHNQQSSQTPTTTGCSGGETNCYKKRWRDHRGYRTERGCGCPSVKNGIEINCCTTDRCNN	Pain		[29]
Exenatide	*Heloderma suspectum*	Synthetic	HGEGTFTSDLSKQMEEEAVRLFIEWLKNGGPSSGAPPPS	Type 2 diabetes mellitus	Amylin	[30,31]
Lixisenatide	*Heloderma suspectum*	Synthetic	HGEGTFTSDLSKQMEEEAVRLFIEWLKNGGPSSGAPPSKKKKKK	Type 2 diabetes mellitus	Sanofi Aventis and Zealand	[32]
Bivalirudin	*Hirudo medicinalis*	Synthetic	FPRPGGGGNGDFEEIPEEYL	Anticoagulant	Biogen	[33]
Desirudin	*Hirudo medicinalis*	Recombinant	VVYTDCTESGQNLCLCEGSNVCGQGNKCILGSDGEKNQCVTGEGTPKPQSHNDGDFEEIPEEYLQ	Prevention of venous thrombotic events		[34]
Lepirudin	*Hirudo medicinalis*	Recombinant	LTYTDCTESGQNLCLCEGSNVCGQGNKCILGSDGEKNQCVTGEGTPKPQSHNDGDFEEIPEEYLQ	Heparin-induced thrombocytopenia		[34]
Ziconotide	*Conus magus*	Synthetic	CKGKGAKCSRLMYDCCTGSCRSGKC	Severe chronic pain	Elan	[35]

**Table 2:** Analgesic drugs that target VGSCs for the treatment of pain.

	VGSC Blockers	Targets	Classification	Indication	Source	References
Marketed drugs	Carbamazepine	VGSC	Antiepileptic drugs	Neuropathic pain	Directly synthesized dibenzazepine family drugs	[55]
	Lidocaine	VGSC	Local anesthetics	Neuropathic pain, Postoperative pain	The analogs of isogarmine	[56]
	Procaine	VGSC	Local anesthetics	Neuropathic pain, Postoperative pain	The structural derivatives of the alkaloid cocaine isolated from the coca plant	[57]
	Oxcarbazepine	VGSC	Antiepileptic drugs	Neuropathic pain	The 10-position ketone derivative of carbamazepine	[58]
	Quinidine	VGSC	Antiarrhythmic drugs	Neuropathic pain	*Cinchona* bark	[59]
Drugs in Research	AZD3161	Nav1.7	Nav1.7 blockers	Neuropathic pain, Inflammatory pain	Artificial design synthesis	[63]
	CNV1014802	Nav1.7	Nav1.7 blockers	Trigeminal neuralgia	Compounds designed based on pyrrolidine	[63,64]
	PF-05089771	Nav1.7	Nav1.7 blockers	Neuropathic pain	Compounds designed based on aryl sulfonamide	[63]
	PF-04856264	Nav1.7	Nav1.7 blockers	Osteoarthritis	Compounds designed based on aryl sulfonamide	[64]
	XEN402	Nav1.7	Nav1.7 blockers	Erythromelalgia	Compounds designed based on pyrrolidine	[65]
	VX-548	Nav1.8	Nav1.8 blockers	Acute pain, Neuropathic pain	Compounds designed based on pyridone amide	[66]
	PF-04531083	Nav1.8	Nav1.8 blockers	Neuropathic pain	Compounds designed based on phenyl imidazole	[63]
	VX-150	Nav1.8	Nav1.8 blockers	Various pain indications	Compounds designed based on pyridone amide	[67]
	2j	Nav1.8	Nav1.8 blockers	Neuropathic pain, Inflammatory pain	The derivative compounds of VX-150	[67]
	Lacosamide	Nav1.3, Nav1.7, Nav1.8	Non-selective sodium channel blockers	Neuropathic pain	Functionalized amino acid	[68]
	Vixotrigine	VGSC	Non-selective sodium channel blockers	Neuropathic pain	Compounds designed based on pyrrolidine	[69,70]

**Table 3:** Characteristics and antibacterial activity of scorpion venom peptides.

	Scorpion Venom Peptides	Sequences	Classification	pI and Net Charge ^a^	Pathogens	MIC and Hemolysis	Species ^b^/ References
Natural peptide	TtAP-1	FLGSLFSIGSKLLPGVFKLFSRKKQ	NDBP	pI = 11.3, +6	*S. aureus*, *S. epidermidis*, *E. faecalis*, *E. faecium*, *C. difficile*, *E. coli*, *P. aeruginosa*, *A. baumannii*, *K. pneumoniae*	3.13–12.5 µg/mL, LC_50_ = 18 µg/mL	TT [109]
	Im-5	FLGSLFSIGSKLLPGVIKLFQRKKQ	NDBP	pI = 11.3, +6	*S. aureus*, *E. coli*, *B. subtilis*	0.5–10 µM, EC_50_ = 28 µM	IM [110]
	UyCT3	ILSAIWSGIKSLF	NDBP	pI = 8.8, +2	*S. aureus*, *E. coli*, *P. aeruginosa*	6–15 µM, 95% hemolysis at 50 µM	UY [111]
	UyCT5	IWSAIWSGIKGLL	NDBP	pI = 8.8, +2	*S. aureus*, *E. coli*, *P. aeruginosa*	1–15 µM, 93% hemolysis at 50 µM	UY [111]
	Pantinin-3	FLSTIWNGIKSLL	NDBP	pI = 9.69, +1	*S. aureus*, *B. megaterium*, *M. luteus*, MRSA, *E. coli*, *K. oxytoca*, *S. enterica*, *CT*	4–87 µM, 70% hemolysis at 16 µM, 100% hemolysis at 32 µM	PI [112]
	Meucin-18	FFGHLFKLATKIIPSLFQ	NDBP	pI = 10, +2	*Bacillus sp*. *DM-1*, *M. Luteus*, *B. megaterium*, *A. tumerfaciens*, *E.coli*, *S. typhimurium*, *S. oneidensis*, *Stenotrophomonus sp*. YC-1, *A. fumigatus*, *G. candidum*, *N. crassa*, *C. albicans*, *S. cerevisiae*, *Beauveria* spp.	0.25–25.1 µM, 74% hemolysis at 6.25 µM	ME [113]
	VmCT1	FLGALWNVAKSVF	NDBP	pI = 8.8, +2	*S. aureus*, *E. coli*, *P. aeruginosa*, *B. subtilis*, *S. typhi*, *S. agalactiae*	5–25µM, 12% hemolysis at 50 µM	VMS [114,115]
	BmKn2	FIGAIARLLSKIF	NDBP	pI = 11, +2	*S. aureus*, *M. luteus*, *B. subtilis*, *E. coli*, *P. aeruginosa*	0.6–21.3 µg/mL, HC_50_ = 17.13 µg/mL, 91.8% hemolysis at 25 µg/mL	BMK [116,117,118]
	Hp1404	GILGKLWEGVKSIF	NDBP	pI = 8.6, +2	*S. aureus*, *S. epidermidis*, *M. luteus*, *B. subtillis*, MRSA, *E. faecium*, *S. agalactiae*	6.25–25 µg/mL, (4.04–16.16 µM) HC_50_ = 226.6 µg/mL (146.5 µM)	HP [116]
	Bactridine 1	KDGYIIEHRGCKYSCFFGTNSWCNTECTLKKGSSGYCAWPACWCYGLPDNVKIFDSNNLKC	DBP	pI = 8.2, +3	*B. subtilis*, *M. luteus*, *E. faecalis*, *Y. enterocolitica*, *P. aeruginosa*, *A. calcoaceticus*	22–77 µM, 0.2% hemolysis at 90 µM, 1% hemolysis at 180 µM	TD [129]
	Heteroscorpine-1	GWINEEKIQKKIDEKIGNNILGGMAKAVVHKLAKGEFQCVANIDTMGNCETHCQKTSGEKGFCHGTKCKCGKPLSY	DBP	pI = 8.8, +7	*S. aureus*, *P. aeruginosa*		HL [130]
Partially derived peptides	Kn2-7	FIKRIARLLRKIF	NDBP	pI = 12.3, +6	*S. aureus*, *E. coli*	5–10 µg/mL, 6.9% hemolysis at 25 µg/mL, 41% hemolysis at 100 µg/mL	[119]
	Kn2-7K	FIKKIAKLLKKIF	NDBP	pI = 10.6, +6	*S. aureus*, *E. coli*	5 µg/mL, 12.2% hemolysis at 100 µg/mL	[119]
	Hp1404-T1e	ILKKLLKKVKKI	NDBP	pI = 10.7, +6	*P. aeruginosa*	0.78–12.5 µM	[120]
	CeHS-1	GWINEEKIQKKIDEKIGNNILGGMAKAVVHKLAKGEFQ	NDBP	pI = 9.2, +2	*K. pneumoniae*, *E. coli*, *B. subtilis*, *S. aureus*, *P. aeruginosa*, *resistant K. pneumoniae*, MRSA	16–128 µg/mL	[131]
	CeHS-1 GP	GWINKIQKKIEKIGNNILGGMAKAGPVVHKLAKGEFQ	NDBP	pI = 10.1, +5	*K. pneumoniae*, *E. coli*, *B. subtilis*, *S. aureus*, *P. aeruginosa*, *resistant K. pneumoniae*, MRSA	16–128 µg/mL	[131]
	CeHS-1 GPK	GWINKIQKKIEKIGNKILGGMAKAGPVVHKLAKGEFQ	NDBP	pI = 10.2, +6	*K. pneumoniae*, *E. coli*, *B. subtilis*, *S. aureus*, *P. aeruginosa*, *resistant K. pneumoniae*, MRSA	8–128 µg/mL	[131]

^a^ The pI values and net charge data are sourced from Heliquest (https://heliquest.ipmc.cnrs.fr/cgi-bin/ComputParams.py, accessed on 4 February 2025), the Antimicrobial Peptide Database APD3 (https://aps.unmc.edu/database/peptide, accessed on 4 February 2025), and relevant literature. ^b^ TT: *Tityus trinitatis*; IM: *Isometrus maculatus*; UY: *Urodacus yaschenkoi*; PI: *Pandinus imperator*; ME: *Mesobuthus eupeus*; VMS: *Vaejovis mexicanus smithi*; BMK: *Buthus martensii Karsch*; HP: *Heterometrus petersii*; TD: *Tityus disrepans;* HL: *Heterometrus laoticus*.

**Table 4:** Characteristics and anticancer activity of scorpion venom peptides.

Scorpion Venom Peptides	Sequences	Classification	Cancer Cells	Modes of Action	Species ^a^/ References
BmK AGAP	VRDGYIADDKNCAYFCGRNAYCDDECKKNGAESGYCQWAGVYGNACWCYKLPDKVPIRVPGKCNGG	DBP	Anti-breast cancer (MCF-7, MDA-MB-231)	Inhibits cell invasion and migration	BMK [133]
Margatoxin	TIINVKCTSPKQCLPPCKAQFGQSAGAKCMNGKCKCYPH	DBP	Anti-lung cancer (A549), anti-prostate cancer (AT-2)	Inhibits cell proliferation	CM [134,135,136]
BmKCT	CGPCFTTDANMARKCRECCGGIGKCFGPQCLCNRI	DBP	Anti-glioma (C6 and U251)	Inhibits cell proliferation and invasion, induces apoptosis	BMK [137,138,139]
Smp24	IWSFLIKAATKLLPSLFGGGKKDS	NDBP	Anti-leukemia cells (KG1-a and CCRF-CEM), anti-lung cancer (A549, H3122, PC-9, and H460), anti-liver cancer (HepG2)	Induces cell necrosis, induces cellular pyroptosis, inhibits cell migration	SMP [140,141,142,143]
Smp43	GVWDWIKKTAGKIWNSEPVKALKSQALNAAKNFVAEKIGATPS	NDBP	Anti-liver cancer (HepG2), anti-lung cancer (A549)	Inhibits cell growth	SMP [144]
Pantinin-1	GILGKLWEGFKSIV	NDBP	Anti-breast cancer (MDA-MB-231), anti-prostate cancer (DU-145)	Induces apoptosis	PI [145]
Pantinin-2	IFGAIWKGISSLL	NDBP	Anti-breast cancer (MDA-MB-231), anti-prostate cancer (DU-145)	Induces apoptosis	PI [145]
Pantinin-3	FLSTIWNGIKSLL	NDBP	Anti-breast cancer (MDA-MB-231), anti-prostate cancer (DU-145)	Induces apoptosis	PI [145]
TsAP-1	FLSLIPSLVGGSISAFK	NDBP	Anti-lung cancer (NCI-HI57, NCI-H838)	Inhibits cell proliferation	TSE [146]
TsAP-2	FLGMIPGLIGGLISAFK	NDBP	Anti-lung cancer (NCI-HI57, NCI-H838), anti-prostate cancer (PC3), anti-breast cancer (MCF-7), anti-glioma (U251)	Inhibits cell proliferation	TSE [146]
AaeAP1a	FLFKLIPKVIKGLVKAIRK	NDBP	Anti-prostate cancer (PC3), anti-lung cancer (NCI-H460), anti-breast cancer (MDA-MB-435S and MCF-7)	Inhibits cell proliferation	AAE [147]
AaeAP2a	FLFKLIPKAIKGLVKAIRK	NDBP	Anti-prostate cancer (PC3), anti-lung cancer (NCI-H460), anti-breast cancer (MDA-MB-435S and MCF-7)	Inhibits cell proliferation	AAE [147]

^a^ CM: Centruroides margaritatus; SMP: Scorpio Maurus palmatus; TSE: Tityus serrulatus; AAE: Androctonus aeneas.

**Table 5:** Characteristics and anticoagulant effects of scorpion venom peptides.

Scorpion Venom Peptides	Sequences	Species ^a^/ References
TanP	YPASFDDDFDALDDLDDLDLDDLLDLEPADLVLLDMWANMMDSQDFEDFE	TST [148]
P8(HA18-3-B-8)	VEPVTVNPHE	BMK [149]
LeuTrp	LW	HL [150]
IIeTrp	IW	HL [150]

^a^ TST: Tityus stigmurus.

## Data Availability

No new data were created or analyzed in this study.

## Appendix A

**Table A1:** Scorpion venom peptides acting on VGSCs exhibit analgesic activity.

Scorpion Venom Peptides	Sequences ^a^	Targets	Animal Pain Models	Accession Numbers ^b^	Species ^c^/ References
Makatoxin-3	GRDAYIAKKEN**C**TYF**C**ALNQY**C**NDL**C**TKNGAKSGY**C**QWAGRYGNA**C**W**C**IDLPDKVPIRIPGP**C**IGR	Nav1.7	Formalin test, acetic acid writhing test, and CFA-induced inflammatory pain model	P59853	BMK [73,74]
ANEP	DGYIRGSNG**C**KIS**C**LWGNEG**C**NKE**C**KGFGAYYGY**C**WTWGLA**C**W**C**EGLPDDKTWKSESNT**C**GGKK	Nav1.7	Acetic acid writhing test	Q9BKJ0	BMK [75]
DKK-SP2	VRDAYIAKPEN**C**VYE**C**AKNEY**C**NDL**C**TKNGAKSGY**C**QWLGKYGNG**C**W**C**IELPDNVPIRVPGK**C**QR	Nav1.7	Acetic acid writhing test		BMK [76]
BmKBTx	DDDPGNYPTNAYGNKYY**C**TILGENEY**C**RKI**C**KLHGVTYGY**C**YNSR**C**W**C**EKLEDKDVTI	Nav 1.7	Acetic acid writhing test		BMK [77,78]
BmNaL-3SS2	VKDRFLIINGSYEL**C**VYAENLGED**C**ENL**C**KQQKATDGF**C**RQPH**C**F**C**TDMPDDYATRPDTVDPIM	Nav 1.7	Acetic acid writhing test		BMK [78]
Syb-prII	not mentioned	Nav1.8	Formalin test		BMK [79,80]
BmK AS	DNGYLLDKYTG**C**KVW**C**VINNES**C**NSE**C**KIRGGYYGY**C**YFWKLA**C**F**C**QGARKSELWNYNTNK**C**NGKL	TTX-R and TTX-S Nav	Formalin test	Q9UAC9	BMK [81,82]
BmK AS-1	DNGYLLNKYTG**C**KIW**C**VINNES**C**NSE**C**KLRRGNYGY**C**YFWKLA**C**Y**C**EGAPKSELWAYETNK**C**DGKL	TTX-R and TTX-S Nav	Heat radiation method	Q9UAC8	BMK [82,83]
BmK IT2	DGYIKGKSGCRVACLIGNQGCLKDCRAYGASYGYCWTWGLACWCEGLPDNKTWKSESNTCG	TTX-R and TTX-S Nav	Formalin test	P68727	BMK [84]
BmK M9	VRDAYIAKPENCVYHCATNEGCNKLCTDNGAESGYCQWGGRYGNACWCIKLPDRVPIRVPGKCH	Nav1.7 Nav1.4 Nav1.5		P45698	BMK [85]
BmK AGAP	VRDGYIADDKN**C**AYF**C**GRNAY**C**DDE**C**KKNGAESGY**C**QWAGVYGNA**C**W**C**YKLPDKVPIRVPGK**C**NGG	Nav1.7 Nav1.8 Nav1.4 Nav1.5	Acetic acid writhing test, hot-plate test	AAP34332.1	BMK [86,87,88]
BmK AGP-SYPU1	GRDAYIAQNYN**C**VYH**C**FRDDY**C**NGL**C**TENGADSGY**C**YLAGKYGHA**C**W**C**INLPDDKPIRIPGK**C**HRR	Nav1.7 Nav1.4 Nav1.5	Acetic acid writhing test	E7CAU3	BMK [89,90,91,92]
BmK IT-AP	KKNGYAVDSSGKVAECLFNNYCNNECTKVYYADKGYCCLLKCYCFGLADDKPVLDIWDSTKNYCDVQIIDLS	ND	Acetic acid writhing test	O77091	BMK [93]
BmK dITAP3	DGYIRGSNG**C**KVS**C**LWGNEG**C**NKE**C**RAYGASYGY**C**WTWGLA**C**W**C**EGLPDDKTWKSESNT**C**G	ND	Acetic acid writhing test	Q9XY87	BMK [94]
BmK AngM1	VRDAYIAKPENCVYECGITQDCNKLCTENGAESGYCQWGGKYGNACWCIKLPDSVPIRVPGKCQR	Nav	Acetic acid writhing test	O61705	BMK [96]
BmK I1	not mentioned	ND	Acetic acid writhing test		BMK [97]
BmK I4	KKNGYAVDSSGKVAECLFNNYCNNECTKVYYADKGYCCLLKCYCFGLADDKPVLDIWDSTKNYCDVQIIDLS	ND	Acetic acid writhing test		BMK [97]
BmK I6	not mentioned	ND	Acetic acid writhing test		BMK [97]
BmK 9	VRDAYIAKPENCVYHCATNEGCNKLCTDNGAESGYCQWGGRYGNACWCIKLPDSVPIRVPGKCHR	ND	Acetic acid writhing test		BMK [98]
BmK AGAP-SYPU2	VKDGYIVDDKNCAYFCGRNAYCDDECEKNGAESGYCQWAGVYGNACWCYKLPDKVPIRVPGRCNG	Nav	Acetic acid writhing test, hot-plate test	G4V3T9	BMK [99]
BmK AGP-SYPU2	VKDGYIADDRNCPYFCGRNAYCDGECKKNRAESGYCQWASKYGNACWCYKLPDDARIMKPGRCNGG	Nav	Acetic acid writhing test	Q9NJC7	BMK [100,101]
Amm VIII	LKDGYIVNDINCTYFCGRNAYCNELCIKLKGESGYCQWASPYGNSCYCYKLPDHVRTKGPGRCND	ND	Hot-plate pain test, tail-flick test	Q7YXD3	AMM [102,103]
LqqIT2	DGYIRKRDG**C**KLS**C**LFGNEG**C**NKE**C**KSYGGSYGY**C**WTWGLA**C**W**C**EGLPDEKTWKSETNT**C**G	ND	Hot-plate pain test, tail-flick test	P19855	LQQ [102]
BotAF	DNGYVLDKYNGCKVCQVINNEACNSECKSRGGYYGYCYFWKLACYCEGANKSELWQYKTNRCRA	ND	Acetic acid writhing test, Formalin test, hot-plate test, tail-flick test		BOT [104]
Cell8	KKDGYPVNMEECRYNCWKNAYCDKLCKEKKGQSGYCYGWNLSCWCIGLPDNTNTKMNPFCQTAD	Nav1.7	ND		CE [105]
IMe-AGAP	VRDGYIADDKN**C**AYF**C**GRNAY**C**DEE**C**KKNGAESGY**C**QWAGQYGNA**C**W**C**YNLPDKVPIKVPGK**C**NGG	Nav1.8 Nav1.9	ND		ME [106]

^a^ A disulfide bond can be formed between two cysteine residues with the same color in the scorpion venom peptide’s amino acid sequence. The cysteine residues are represented in red, blue, green, and orange. ^b^ The accession numbers for scorpion venom peptides were obtained from the UniProt database (https://www.uniprot.org/, accessed on 4 February 2025). ^c^ AMM: *Androctonus mauretanicus mauretanicus*; LQQ: *Leiurus quinquestriatus quinquestriatus*; BOT: *Buthus occitanus tunetanus*; CE: *Centruroides elegans.*

**Table A2:** List of forms.

Scorpion Venom Peptides	Biological Activity	References
Makatoxin-3	Analgesic, targeting Nav1.7 channel	[73,74]
ANEP	Analgesic, targeting Nav1.7 channel	[75]
DKK-SP2	Analgesic, targeting Nav1.7 channel	[76]
BmKBTx	Analgesic, targeting Nav1.7 channel	[77,78]
BmNaL-3SS2	Analgesic, targeting Nav1.7 channel	[78]
Syb-prII	Analgesic, targeting Nav1.8 channel	[79,80]
BmK AS	Analgesic, targeting TTX-R and TTX-S Nav channels	[81,82]
BmK AS-1	Analgesic, targeting TTX-R and TTX-S Nav channels	[82,83]
BmK IT2	Analgesic, targeting TTX-R and TTX-S Nav channels	[84]
BmK M9	Analgesic, targeting Nav1.7, Nav1.4, and Nav1.5 channels	[85]
BmK AGAP	Analgesic, anticancer, targeting Nav1.7, Nav1.8, Nav1.4, and Nav1.5 channels	[86,87,88]
BmK AGP-SYPU1	Analgesic, targeting Nav1.7, Nav1.4, and Nav1.5 channels	[89,90,91,92]
BmK IT-AP	Analgesic	[93]
BmK dITAP3	Analgesic	[94]
BmK AngM1	Analgesic	[96]
BmK I1	Analgesic	[97]
BmK I4	Analgesic	[97]
BmK I6	Analgesic	[97]
BmK 9	Analgesic	[98]
BmK AGAP-SYPU2	Analgesic, anticancer	[99]
BmK AGP-SYPU2	Analgesic	[100,101]
Amm VIII	Analgesic	[102,103]
LqqIT2	Analgesic	[102]
BotAF	Analgesic	[104]
Cell8	Targeting Nav1.7	[105]
IMe-AGAP	Targeting Nav1.7 and Nav1.8, anticancer	[106,151]
TtAP-1	Antibacterial	[109]
Im-5	Antibacterial	[110]
UyCT3	Antibacterial	[111]
UyCT5	Antibacterial	[111]
Pantinin-3	Antibacterial, anticancer	[112]
Meucin-18	Antibacterial	[113]
VmCT1	Antibacterial, anticancer	[114,115,152]
BmKn2	Antibacterial, anticancer	[116,117,118]
Hp1404	Antibacterial	[116]
Bactridine 1	Antibacterial	[129]
Heteroscorpine-1	Antibacterial	[130]
Kn2-7	Antibacterial	[119]
Kn2-7K	Antibacterial	[119]
Hp1404-T1e	Antibacterial	[120]
CeHS-1	Antibacterial	[131]
CeHS-1 GP	Antibacterial	[131]
CeHS-1 GPK	Antibacterial	[131]
Margatoxin	Anticancer, non-selective Kv1.3 inhibitor	[134,135,136,153]
BmKCT	Anticancer	[137,138,139]
Smp24	Anticancer, antibacterial	[140,141,142,143]
Smp43	Anticancer, antibacterial	[144]
Pantinin-1	Anticancer, antibacterial	[112,145]
Pantinin-2	Anticancer, antibacterial	[112,145]
Pantinin-3	Anticancer, antibacterial	[112,145]
TsAP-1	Anticancer, antibacterial	[146]
TsAP-2	Anticancer, antibacterial	[146]
AaeAP1a	Anticancer, antibacterial	[147]
AaeAP2a	Anticancer, antibacterial	[147]
TanP	Anticoagulant	[148]
P8(HA18-3-B-8)	Anticoagulant	[149]
LeuTrp	Anticoagulant	[150]
IIeTrp	Anticoagulant	[150]
